# Disseminated infection with *Nocardia otitidiscaviarum* in a patient under steroid therapy

**DOI:** 10.1002/ccr3.2640

**Published:** 2020-01-07

**Authors:** Ranjit Sah, Shusila Khadka, Samikshya Neupane, Gaurav Nepal, Sonam Singla, Pankhuri Kumari, Sanjit Sah, Ranjana Sah, Shyam Sundar Sah, Mahesh Adhikari, Niranjan Prasad Shah, Bharat Mani Pokharel, Basista Rijal, Dibya Singh Shah

**Affiliations:** ^1^ Tribhuvan University Institute of Medicine Kathmandu Nepal; ^2^ Medanta The Medicity Gurgaon India

**Keywords:** nephrotic syndrome, *Nocardia otitidiscaviarum*, pulmonary nocardiosis, steroid therapy, subcutaneous abscess

## Abstract

Corticosteroid recipients with lung infections should be suspected of having nocardiosis; however, nocardiosis can easily mimic malignancy, tuberculosis, or fungal infection. Though cultural identification is possible, it might be missed due to its slow growth pattern.. Therefore, if filamentous bacteria are seen during staining, plate incubation time should be extended.

## Graphical abstract

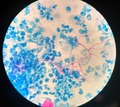

## 1 BACKGROUND

*Nocardia otitidiscaviarum* is a rare pathogen and is known to be less pathogenic than other *Nocardia* species.

The genus *Nocardia* is a ubiquitous group of environmental bacteria found in soil, decomposing vegetation, and other organic matter, as well as in fresh and salt water.^1^ It usually manifests as an opportunistic infection in immunocompromised hosts. It is Gram‐variable, acid‐fast, and branching filamentous bacteria which grows aerobically. In humans, the infection occurs either by inhalation of dust or contamination of wound or by deep implantation. Hence, *Nocardia* can cause pulmonary, superficial cutaneous, and subcutaneous infection (mycetoma).^2^

*Nocardia* infection can be caused by various species, including *N asteroides*, *N brasiliensis*, *N otitidiscaviarum*, *N cyriacigeorgica,* and *N farcinica*.^3^ Out of all *Nocardia* species, *N asteroids*, *N farcinica*, and *N brasiliensis* are the primary pathogens causing nocardiosis, while other species are rare or reported less frequently.^4^ *N otitidiscaviarum* rarely causes infection, and it was first recognized in samples taken from a Sumatran cavy or guinea pig with ear disease.^3^ It is usually less pathogenic than other species of *Nocardia*.^5^, ^6^

Herein, we present a case of *N otitidiscaviarum*, a rare and less pathogenic organism causing severe pulmonary and lymphocutaneous (subcutaneous abscess) infection.

## 2 CASE PRESENTATION

A 61‐year‐old man presented to Tribhuvan University Teaching Hospital (TUTH), Kathmandu, Nepal, with chief complaints of fever for seven days along with coughing and swelling of the right thigh. Fever was continuous, with maximum recorded temperature of 102 degree Fahrenheit. There was no history of night sweats, hemoptysis, weight loss or abdominal swelling. The patient did not report any history of trauma. There was no contact history with tuberculosis patient. For his illness, he was treated with intravenous ceftriaxone at a local hospital for 10 days; however, his symptoms did not improve and was referred to TUTH.

He was on steroid (1 mg/kg/d) therapy since 8 weeks for his recent diagnosis of nephrotic syndrome (focal segmental glomerulosclerosis) on renal biopsy. On clinical examination, a mass was palpated over his right thigh. His cardiovascular, neurological, and gastrointestinal examinations were unremarkable. On respiratory examination, patient was tachypneic, dull note was heard over right upper hemithorax, and breath sound was deceased on right upper hemithorax.

**Figure 1:**
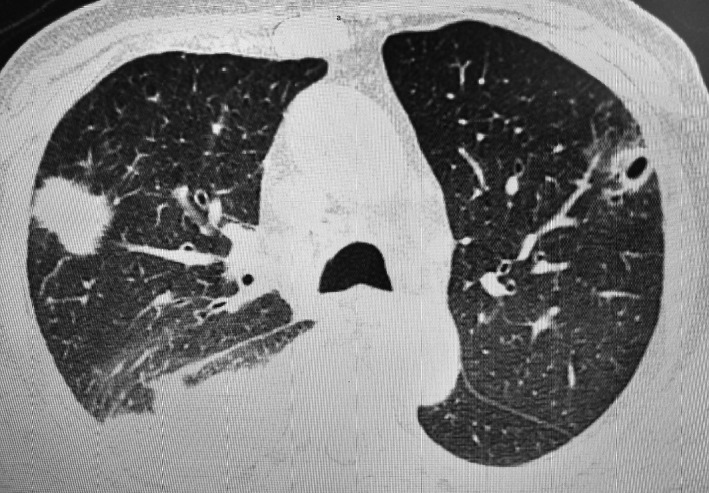
HRCT of chest showed consolidation (mass‐like lesion 3.5 × 3.5 cm) in the right upper lobe with right‐sided pleural effusion and cystic lesion in the left upper lobe

On laboratory examination, his total white blood cell (WBC) count was 16 000/µL with neutrophilic predominance (82%). Erythrocyte sedimentation rate (>56 mm/h) and C‐reactive protein (112 mg/L) were elevated with normal procalcitonin (0.4 ng/mL) level. His serum creatinine (0.9 mg/dL) and liver enzymes were within normal range. Serum markers for HIV, hepatitis B and hepatitis C, and syphilis were negative. Chest X‐ray showed opacities. High‐resolution computed tomography (HRCT) of chest showed consolidation (mass‐like lesion 3.5 × 3.5 cm) in the right upper lobe with right‐sided pleural effusion and cystic lesion in the left upper lobe (Figure 1). MRI of the brain revealed no neurological findings. Ultrasonography (USG) of the abdomen was normal but USG of thigh showed pus collection in the right thigh.

**Figure 2:**
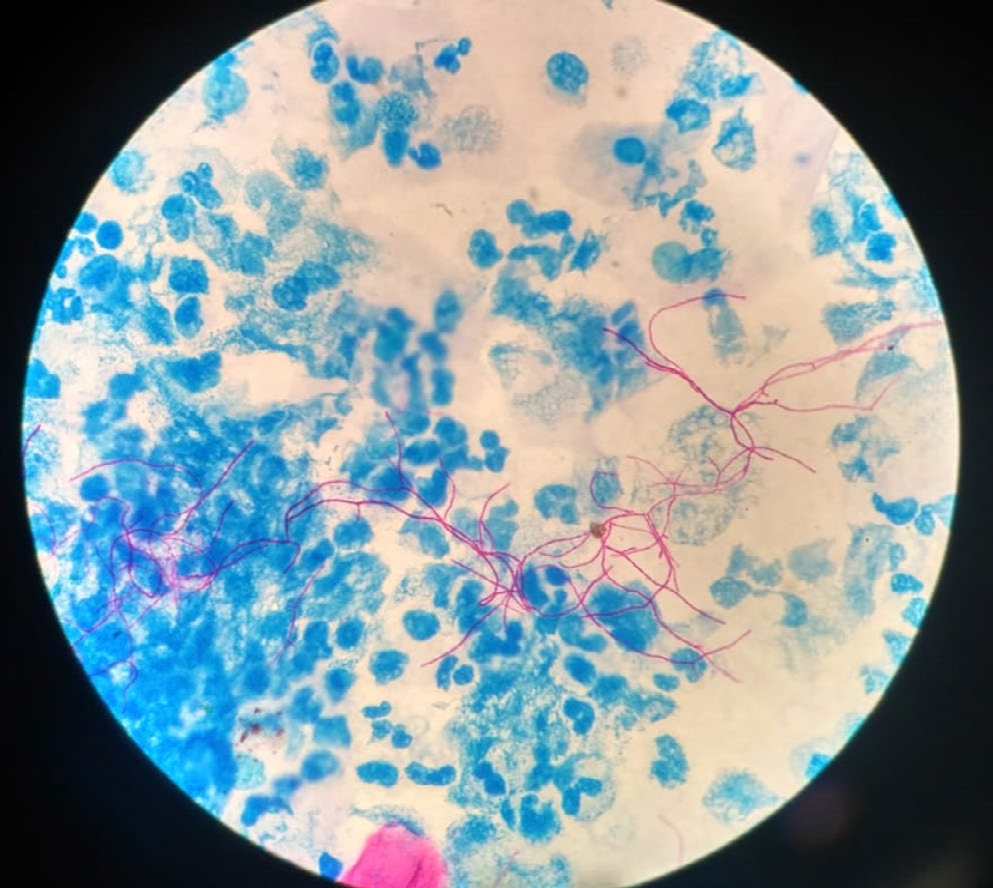
Modified Ziehl‐Neelsen (ZN) stain of sputum showing acid‐fast branching filamentous organism

**Figure 3:**
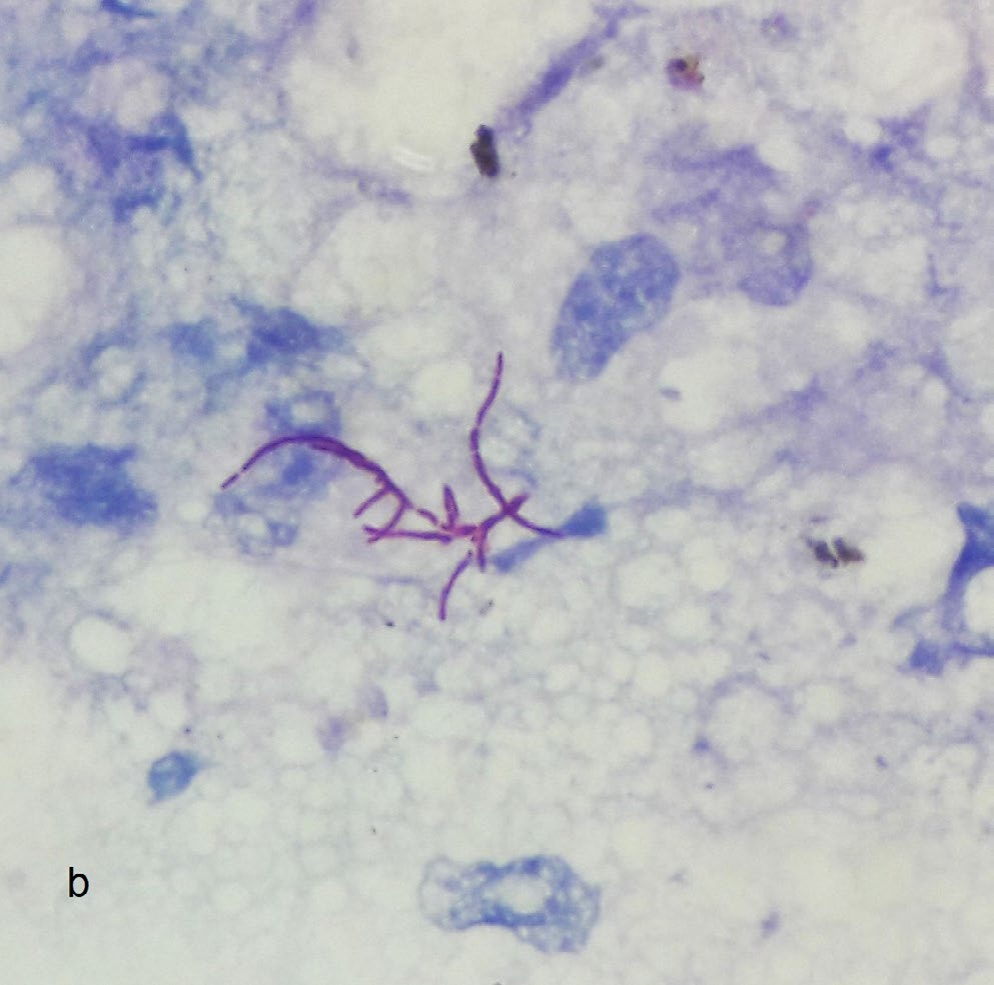
Modified Ziehl‐Neelsen (ZN) stain of pus showing acid‐fast branching filamentous organism with beaded appearance

**Figure 4:**
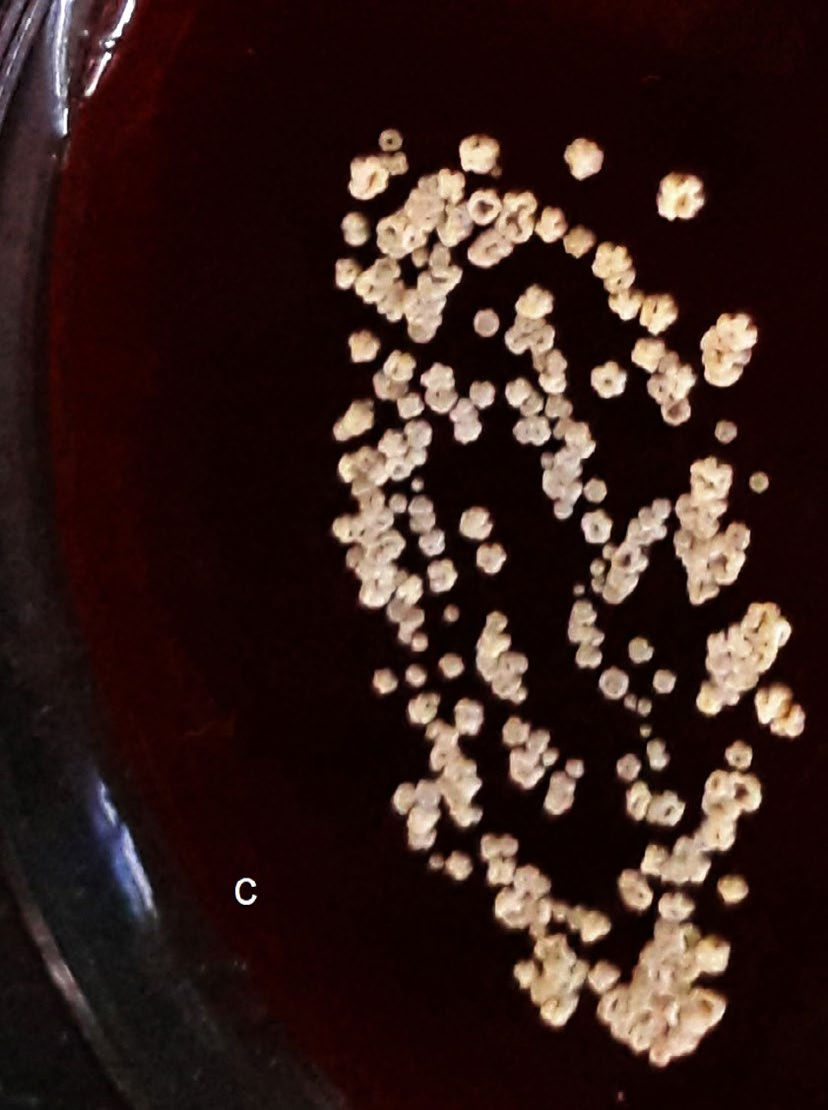
Molar tooth appearance colonies of *Nocardia otitidiscaviarum* on blood agar at prolonged incubation

Gram staining of sputum sample showed Gram‐variable branching filamentous bacteria. Modified Ziehl‐Neelsen (ZN) staining of the same sample showed acid‐fast branching filamentous bacteria (Figure 2). Gene Xpert MTB/RIF test of sputum sample was negative. On aerobic culture, chalky‐white colonies were seen on blood agar. The subcutaneous abscess was aspirated, and the pus was sent to laboratory for further examination. Gram stain revealed plenty of pus cells, with Gram‐variable, branching, and filamentous bacteria. On the background of filamentous bacteria on Gram stain and ZN stain, modified ZN stain was performed. It revealed numerous acid‐fast branching filamentous organism with beaded appearance (Figure 3). The pus was cultured both aerobically on blood and MacConkey agar and anaerobically in Robertson's cooked meat media and incubated at 37°C. There was no growth in the agar plates at 24 hours of incubation, and no turbidity was seen in Robertson's cooked meat media. But on further incubation, adherent chalky‐white colonies were seen on blood agar. On prolonged incubation, the colonies became yellowish and had molar tooth appearance (Figure 4). These colonies were positive with catalase test and urea hydrolysis test. However, no growth was seen on MacConkey agar and in Robertson's cooked meat media which was incubated anaerobically.

For species identification, the isolated colonies were transported to India where VITEK^®^ MS was applied which uses matrix‐assisted laser desorption/ionization time‐of‐flight (MALDI‐TOF) technology. As a result, *Nocardia otitidiscaviarum* was identified with confidence value of 99.9%. The colonies were processed for antimicrobial susceptibility testing by microbroth dilution method as per CLSI guidelines and were sensitive to cotrimoxazole, imipenem, amikacin, and linezolid but resistance to ceftriaxone.

His respiratory distress increased, leading to high oxygen demand. Therefore, he was shifted to ICU and intubated. His weight was 58 kg, height 5.7″, and BMI 19.9. So meropenem (1 gm three times daily) and amikacin (750 mg once daily) were initiated in addition to cotrimoxazole. Arterial blood gas analysis revealed pH—7.491, Pao2—51.9 mm Hg, Pco2—30.5 mm Hg, and Hco3—23.5 mmol/L with Pao2/Fio2—86.4 mm Hg. After 7 days, his fever resolved with a decrease in WBC count and improvement in respiratory symptoms. He was shifted to general ward where amikacin (750 mg once daily) with cotrimoxazole (800/160 two tab twice daily) was continued and linezolid (600 mg twice daily) was added to the regimen for 21 days as induction therapy. Meropenem was switched to linezolid in view of species identified as *N otitidiscaviarum* which is less susceptible to beta‐lactam antibiotics.

**Figure 5:**
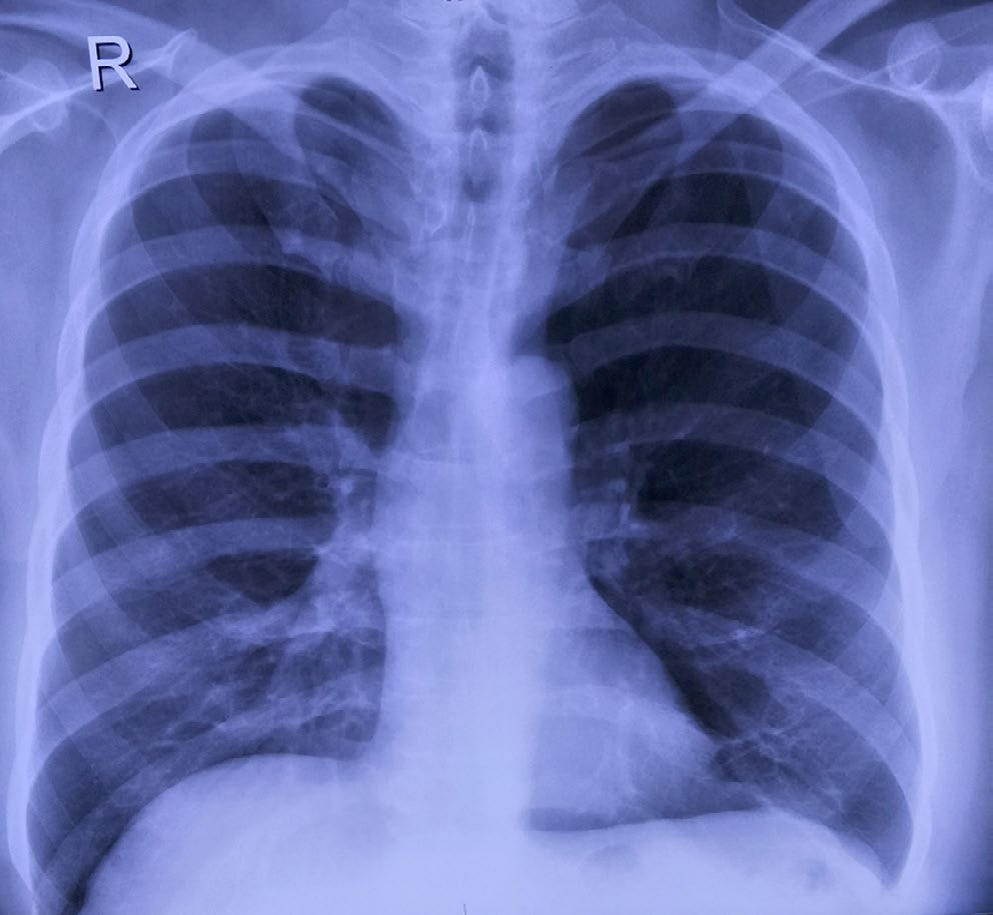
Chest X‐ray at 6 mo showing resolution of the pulmonary lesion

He was discharged with cotrimoxazole for 6 months as maintenance therapy. Chest X‐ray was done at 1, 3, and 6 months (Figure 5) which showed progressive resolution of the lesion. He was on regular follow‐up without any drug reaction or complication. There was no relapse of symptoms at one‐year follow‐up.

## 3 DISCUSSION

To the best of our knowledge, this is the first case of *N otitidiscaviarum* infection in Nepal. Few cases of *Nocardia* infection of brain, lung, eye, and soft tissue have been reported from Nepal but none of them had performed genetic analysis and identification up to species level. Snijders first isolated *N otitidiscaviarum* in 1924,^7^, ^8^ and Gordon et al first identified its biochemical reactions and distinguished it from other *Nocardia* species.^8^ Nomenclature of *N caviae* was originally proposed, but now the organism is called *N otitidiscaviarum*.^8^, ^9^ A study performed at the National Reference Laboratory in Germany from 1979 to 1991 isolated *Nocardia* spp. form 131 patients; however, only 8 of them were infected with *N otitidiscaviarum*.^8^ *N otitidiscaviarum* caused much less infections than other *Nocardia*, which may be due to its lower prevalence in soil and its reduced pathogenicity.^8^ Despite its low prevalence and pathogenicity, sometimes like our case, it can lead to severe infection.

*Nocardia otitidiscaviarum* has been described as an opportunistic pathogen in human.^10^, ^11^ However, it has been reported in both immunocompromised and immunocompetent individuals causing pulmonary, primary cutaneous, and lymphocutaneous infections.^11^, ^12^ Individuals with weak immune system, such as patients suffering from chronic obstructive pulmonary disease, diabetes mellitus, mixed connective tissue disorder, ulcerative colitis, cirrhosis, human immunodeficiency virus infection, malignancies, those receiving long‐term corticosteroid therapy, and bone marrow or solid organ transplant, are at higher risk.^11^, ^13^

*Nocardia* infection has nonspecific pathogenic signs or symptoms, and its clinical picture may mimic a variety of other bacterial infections including actinomycosis, tuberculosis, fungal infections, and malignancies.^14^ A definitive diagnosis can be made by isolation and identification of the organism. For the initial evaluation, Gram stain and modified acid‐fast stain must be done.^15^

Although *Nocardia* spp. grow on ordinary blood agar, but its growth is slow (ranging from 2 days to weeks). Hence, the plates should be incubated for up to 2 or 3 weeks for slowly growing *Nocardia* species.^4^

The drug choice for *Nocardia* infection is cotrimoxazole, but some of the species are resistance to cotrimoxazole, imipenem, amoxicillin‐clavulanic acid, and other antibacterial agents (Table 1) 14, 15, 16, 17, 18, 19, 20, 21, 22; therefore, accurate identification at species level and antimicrobial susceptibility testing must be done.^19^ *N otitidiscaviarum* infection requires treatment for long duration, and also, it is suggested that antibiotic therapy should be continued for 6 months in immunocompetent patients and up to a year in immunosuppressed individuals.^11^

**Table 1:** Antimicrobial susceptibility patterns for common *Nocardia* species^14^, ^15^, ^16^, ^17^, ^18^, ^19^, ^20^, ^21^, ^22^

	*N otitidiscaviarum*	*N Brasiliensis*	*N abscessus*	*N farcinica*	*N nova*	*N cyriacigeorgica*
Trimethoprim‐sulfamethoxazole	Sensitive	Sensitive	Sensitive	Sensitive	Sensitive	Sensitive
Amikacin	Sensitive	Sensitive	Sensitive	Sensitive	Sensitive	Sensitive
Tobramycin	Sensitive	Sensitive	Sensitive	Resistant	Resistant	Sensitive
Linezolid	Sensitive	Sensitive	Sensitive	Sensitive	Sensitive	Sensitive
Imipenem	Resistant	Resistant	Sensitive	Sensitive	Sensitive	Sensitive
Ceftriaxone	Resistant	Resistant	Sensitive	Resistant	Sensitive	Sensitive
Amoxicillin‐clavulanate	Resistant	Sensitive	Sensitive	Sensitive	Resistant	Resistant
Minocycline	Resistant	Resistant	Sensitive	Resistant	Resistant	Resistant
Doxycycline	Resistant	Resistant	Sensitive	Resistant	Resistant	Resistant
Erythromycin	Resistant	Resistant	Resistant	Resistant	Sensitive	Resistant
Clarithromycin	Resistant	Resistant	Resistant	Resistant	Sensitive	Resistant
Ciprofloxacin	Resistant	Resistant	Resistant	Resistant	Resistant	Resistant

Most *N otitidiscaviarum* isolates are resistant to beta‐lactams like imipenem, ampicillin, and amoxicillin‐clavulanic acid but are susceptible to fluoroquinolones and amikacin.^5^, ^11^ Meanwhile, some other studies have shown that *N otitidiscaviarum* complex is sensitive to linezolid in vitro; however, data from in vivo studies are lacking. In addition, 4 weeks of linezolid treatment increases the risk of hematological toxicity, and also, clinical experience with linezolid treatment is limited.^11^, ^20^ In our case, we prescribed 3 weeks of linezolid in combination with cotrimoxazole and amikacin as induction therapy followed by maintenance with cotrimoxazole for six months.

Minocycline can be used as an alternative agent when sulfa drugs cannot be given. Imipenem and amikacinin higher doses have been used in severe or refractory cases.^20^ Other alternative antimicrobial agents include amoxicillin‐clavulanic acid, ceftriaxone, cefotaxime, meropenem, linezolid, moxifloxacin, levofloxacin, and tigecycline can be also used. To minimize the risk of relapse, the treatment is generally prolonged.^1^

The US National Committee of Clinical Laboratory Standards has approved broth microdilution as antimicrobial susceptibility testing for both *Actinomycetes* and *Nocardia*.^11^ This may guide the treatment of *N otitidiscaviarum*, but the optimal treatment protocol of *N otitidiscaviarum* is still unknown. A combination of sulfonamides and amikacin with carbapenem or third generation cephalosporin had been suggested for severe or disseminated infections.^11^ Similarly, in this case, the combination therapy (amikacin, meropenem, and cotrimoxazole) was administered empirically and then modified to amikacin, cotrimoxazole, and linezolid after identification of the species.

*Nocardia otitidiscaviarum* is isolated rarely; however, it can cause localized or disseminated infection, even in an immunocompetent host. The majority of patients responds to cotrimoxazole and aminoglycoside. Identification and drug susceptibility testing for *Nocardia* species are critical for guiding clinical treatment.^8^

## 4 CONCLUSION

Diagnosis of *Nocardia* infection is often misleading and can be initially diagnosed as malignancy or other bacterial (eg, tuberculosis) or fungal infection due to its clinical resemblance. It is usually missed in routine culture because of its slow growth. Therefore, laboratory should keep the culture plate for prolonged incubation whenever filamentous bacteria is seen in Gram stain or modified ZN stain. Physicians should suspect *Nocardia* infection as differential diagnosis in patient receiving steroids with pulmonary lesions. Species identification and antibiotic susceptibility testing are required for guiding antibiotic therapy for *Nocardia* infection, and the patient should be treated with prolonged antibiotic therapy for complete cure and prevention of relapse.

## CONFLICT OF INTEREST

Authors declare: No conflicts of interest. 

Consent to publish: For publication of this case report, written informed consent was taken from the patient.

## AUTHORS' CONTRIBUTIONS

RS established the diagnosis, reviewed the literature, and designed the manuscript. SK, SN, GN, SS, PK, SS, RS, SSS, MA, NPS, BMP, BR, and DSS reviewed the literature and prepared the article. All authors read and approved the final version of the manuscript.

## ETHICAL APPROVAL

According to the local ethical guidelines, ethical approval is not required for a case report.
